# Effective Gamification of the Stop-Signal Task: Two Controlled Laboratory Experiments

**DOI:** 10.2196/17810

**Published:** 2020-09-08

**Authors:** Maximilian Achim Friehs, Martin Dechant, Sarah Vedress, Christian Frings, Regan Lee Mandryk

**Affiliations:** 1 Department of Cognitive Psychology and Methodology Trier University Trier Germany; 2 Human-Computer-Interaction Lab Department of Computer Science University of Saskatchewan Saskatoon, SK Canada

**Keywords:** video games, games, experimental, proof of concept study, cognition, psychology, motivation

## Abstract

**Background:**

A lack of ability to inhibit prepotent responses, or more generally a lack of impulse control, is associated with several disorders such as attention-deficit/hyperactivity disorder and schizophrenia as well as general damage to the prefrontal cortex. A stop-signal task (SST) is a reliable and established measure of response inhibition. However, using the SST as an objective assessment in diagnostic or research-focused settings places significant stress on participants as the task itself requires concentration and cognitive effort and is not particularly engaging. This can lead to decreased motivation to follow task instructions and poor data quality, which can affect assessment efficacy and might increase drop-out rates. Gamification—the application of game-based elements in nongame settings—has shown to improve engaged attention to a cognitive task, thus increasing participant motivation and data quality.

**Objective:**

This study aims to design a gamified SST that improves participants’ engagement and validate this gamified SST against a standard SST.

**Methods:**

We described the design of our gamified SST and reported on 2 separate studies that aim to validate the gamified SST relative to a standard SST. In study 1, a within-subject design was used to compare the performance of the SST and a stop-signal game (SSG). In study 2, we added eye tracking to the procedure to determine if overt attention was affected and aimed to replicate the findings from study 1 in a between-subjects design. Furthermore, in both studies, flow and motivational experiences were measured.

**Results:**

In contrast, the behavioral performance was comparable between the tasks (*P*<.87; BF01=2.87), and the experience of flow and intrinsic motivation were rated higher in the SSG group, although this difference was not significant.

**Conclusions:**

Overall, our findings provide evidence that the gamification of SST is possible and that the SSG is enjoyed more. Thus, when participant engagement is critical, we recommend using the SSG instead of the SST.

## Introduction

### Background

Gamification is the process of applying game design elements (eg, scoring systems, graphical interface, narrative) to nongame environments (eg, cognitive tasks, work context) to increase task performance and engagement [1]. Gamification has been used in a variety of settings, such as in business [2] and education [3]. Serious games are also used in the context of health care education to support desirable behavior [1,4-7]. The use of games or game-like tasks makes it possible to enhance voluntary engagement and decrease participant drop-out rates [8,9]; in fact, a recent study showed that the experience of playing digital games as compared with standard cognitive tasks was perceived as less stressful [10]. A high dropout rate, especially in difficult-to-obtain samples, can lead to difficulties interpreting the results, for example, due to decreased statistical power [11-14]. Increased task engagement is especially important when cognitive tasks are used as a diagnostic tool because they rely upon the participant to perform the task to the best of their ability. Data obtained from individuals who lack the motivation to perform the task will not be representative of their ability, and this can lead to misinterpretations [15-17]. Although it seems that gamification is generally useful, it can also change task performance in an undesired direction [18]. For example, adding a simple scoring or reward system creates a motivational pull that can interact and interfere with the to-be-measured variable and change behavior [19-21]. However, a reward can even capture attention when it is counterproductive to the task performance, which might make simple reward elements, for example, not always suitable for all gamification purposes [22].

### Cognitive Task Gamification

There have been efforts to gamify cognitive tasks for the purposes of training and assessment [23,24]. The interpretation of cognitive task data depends on the assumption that individuals are putting forth their best effort and are fully attentive to the task, but cognitive tasks are often repetitive and boring, so unfocused effort is a common problem [15,16]. An individual’s true ability will not be represented if they are not engaged and fully attentive, which can lead to inaccurate interpretations of cognitive task performance data [17]. To improve engagement with cognitive tasks, researchers have looked to games [25,26], with Aeberhard et al [27] noting that “leveraging gamification to repeatedly obtain behavioral samples paves the way for next-generation high-throughput psychometric toolset.”

However, caution must be taken when introducing game elements to cognitive tasks owing to the risk of muddying the measurement of the targeted cognitive process [28]. Cognitive tasks are very sensitive to manipulation—even basic tasks (eg, Stroop task, dot-probe task) are extensively studied to understand the effects of making small changes to the task paradigm [29]. Adding game-based elements to basic cognitive tasks could affect performance and experience in unintended ways [28]. Studies on how gamification of cognitive tasks affect behavior have shown mixed results. For example, adding points (a common gamification technique) to a task has been shown to increase engagement [28,30-32] and improve performance, such as by facilitating faster reaction times [24,28]. However, the inclusion of points has also shown to increase error rates in a dot-probe task [28]. Adding thematic elements and complex graphics has been shown to lead to decreased performance: for example, in a go-no-go task, the use of cowboy characters resulted in worse performance compared with a control (green and red objects) [31], and the use of zombie characters resulted in worse performance compared with a control (circles and squares) [33], likely because the stimuli were not as simple to discriminate. However, the inclusion of thematic elements and graphical stimuli have been shown to increase enjoyment [31] but also decrease enjoyment [28,30,33], relative to a control task.

As there is little agreement on how typical gamification approaches affect performance on, and engagement with, cognitive tasks [24,28], it is imperative that gamified cognitive tasks, intended for use in research, be validated against the basic version before use. Especially, in the context of cognitive psychology or clinical diagnostics, it is important to maintain internal validity [24,34].

### Theoretical Underpinnings of Gamification

There are many theories that go beyond the mantra of “games are fun” as to why game design elements are so successful in shaping behavior. Although there is still an open debate regarding the understanding of what makes games enjoyable [35], two of the most prominent theories are the Flow Theory of Motivation [36] and the Self-Determination Theory (SDT) [37].

The Flow Theory states that there are some factors that facilitate flow experience. Specifically, the activity must have clear goals, there must be immediate and unambiguous feedback during task performance, and the perceived challenges of the activity must be balanced with the individual’s own skills [38-40]. A flow experience itself differs from individual to individual but is generally characterized by a high concentration on the task at hand, a loss of self-consciousness, a loss of sense of time, and deriving personal purpose from the task performance (ie, autotelic experience) [36,38-40]. In games and player experience research, flow is a key concept and has been proven, among other factors, to be important for player motivation and retention [41-46].

SDT is based upon 3 basic needs: the need for competence (ie, experiencing mastery over challenges); the need for autonomy (ie, doing something owing to an individual’s own volition); and the need for relatedness (ie, experiencing meaningful social relations) [47-49]. Importantly, games have been shown to be capable of addressing those needs and enhancing intrinsic motivation [37]. If one or ideally all 3 needs are satisfied, the motivation to engage in the task will increase [50-53]. SDT has been mirrored in the gamification classification system developed by Nicholson [54], in which he proposed 2 types of gamification: reward-based gamification and meaningful gamification. Although reward-based gamification aims to modify extrinsic motivation, meaningful gamification aims to increase intrinsic motivation. Thus, SDT can be used to explain the underlying components of intrinsic motivation, which has been shown to be an important predictor of task engagement [37,55].

In summary, flow theory and SDT are 2 promising theories that can explain an individual’s motivation for and experience while performing a task. Importantly, the 2 perspectives are not mutually exclusive but rather complement each other. Thus, gamification based on these theories can inform certain design guidelines for developing gamified versions of cognitive tasks. [54,56].

### The Stop-Signal Task

One such cognitive task that is valuable to assess is the ability to inhibit an already initiated action. For example, a basketball player on defense might have to suppress his or her jumping response to avoid falling for the pump-fake of the offensive player or a person might have to stop crossing the street to avoid a speeding car. This type of response inhibition process can be measured using the stop-signal task (SST), which is an established measure of response inhibition and has been used in laboratories now for over 50 years [57,58]. The ability to inhibit a response is also modulated by inter- and intrapersonal differences in humans. For example, a reduction in inhibitory control and a general increase in impulsivity can be seen in people with attention-deficit/hyperactivity disorder (ADHD) [59,60] or patients with schizophrenia [61,62]. In addition, evidence suggests that training or certain types of sports [63,64], as well as noninvasive brain stimulation, can modulate an individual’s ability to stop a response [65,66]. As response inhibition has been consistently associated with certain disorders, it has been proposed that response inhibition capabilities can be used as a form of objective diagnostic indicator, especially in ADHD but also in other disorders such as obsessive-compulsive disorder (OCD) [60,67-69]. Individuals affected by mental disorders, especially in the case of ADHD, may have problems focusing on the cognitive task, which makes it particularly important to develop a task that is more engaging to properly assess their cognitive functioning. However, consideration must be taken as gamified tasks have been shown to normalize the performance of individuals with ADHD, meaning that the gamified cognitive task no longer differentiates between people with and without ADHD [70].

The SST requires the participant to withhold their response on a random subset of trials during a choice reaction time task. The delay after which the stopping cue is presented (aptly termed stop-signal delay [SSD]) is fitted to the individual so that in approximately half of all stopping trials, the response inhibition will fail. In detail, when a participant successfully stops their response, the SSD is increased, making a successful stopping less likely on the subsequent trial (vice versa for unsuccessful stop-trials). Usually, participants are tested in a controlled, distraction-free environment, and the stimuli are presented on a monochromatic screen without any irrelevant or interfering elements. Although this leads to a very precise and clean measurement of an individual’s response inhibition capabilities, it is not comparable with everyday situations in which the stopping of an already initiated response is required.

In other areas dealing with inhibition of information or responses, an effort has been made to transfer fundamental research principles to applied settings. For example, it was shown that the conflict resolution process as measured by classical cognitive psychological tasks such as the Stroop task or Eriksen flanker task [71,72] is conceptually similar and abides by the same rules as deceptive actions in sports [73-75]. Interestingly, recent studies provide evidence that even the underlying neural generators of these 2 conceptually analog tasks are similar [76,77]. However, as previously mentioned, caution must be taken when adding visual complexity to cognitive tasks due to the potential effects on performance.

In the case of the SST, previous work has shown that changing the stimuli from colored circles to colored fruits (along with an accompanying narrative) resulted in greater stop-signal reaction times (SSRTs; ie, worsened performance) relative to a version gamified with points, but no narrative [30]; however, enjoyment was also reduced in the thematic version relative to the points version, suggesting that engagement may not have been facilitated through the particular theme and stimuli chosen. Research on other tasks has suggested that a poorly implemented theme that offers little gameplay might be worse for engagement than including no theme at all [28].

### This Study

To better understand everyday human behavior or, in the case of this paper, specifically stopping the behavior, gamification might be helpful to aid researchers in gathering large data sets over time. In this case, a gamified version of the SST would allow researchers to enhance the ecological validity of the inhibition measurement by presenting the task in a visually complex environment, while also keeping participants motivated to perform well. This ties into the proposition that modern technology can be used to enhance mundane and experimental realism while keeping experimental control high and potentially even increase the effect of experimental manipulation [78,79]. A gamified SST can mirror a more natural setting and therefore elicit more natural responses without sacrificing experimental control. Thus, it is important to choose a task design that not only reliably taps into the targeted processes (ie, the response inhibition process) but also leads to increased participant engagement [80,81]. However, the game must be validated against a basic task to ensure the efficacy of gamification and validity of measurement. In this paper, we present the design of a gamified SST (the stop-signal game [SSG]) and evaluate it relative to the basic task in 2 experiments that consider the effect of gamification on both performance and player experience.

## Methods

### Overall Procedure

This study sets out to evaluate a gamified version of the SST—termed SSG—along the 2 dimensions of performance and experience. We employed 2 studies to show that performance in a standard SST and in the new SSG was comparable within (study 1) and between (study 2) participants. Thus, comparing performance data in study 1 would give insight into the comparability of both tasks without adding unexplained variance in the form of interindividual variability. Study 2 aimed to replicate the results from study 1; a robust result should still hold even for between-group comparisons. Furthermore, we measured motivation and flow using the Intrinsic Motivation Inventory (IMI) [49] and the Flow State Scale (FSS) [82] in both studies to measure participant experience. Finally, in study 2, we employed an eye-tracking protocol to explain the influence of complex graphics on gaze behavior, and ultimately participant performance. We are of the opinion that the risk of sequence and carry-over effects in eye-tracking studies is especially high as participants pay increased attention to their eye movements. Thus, eye tracking was only employed in study 2. The whole eye-tracking procedure was comparable with earlier studies utilizing eye tracking in combination with the SST [83]. This paper aims to show that by leveraging gamification, cognitive tasks can be redesigned to produce more realistic and better data, without compromising internal validity [30,78].

### Study 1: Within-Subject Design Participants

A total of 30 young, healthy adults were recruited for the study (16 female, 13 male, and 1 nonbinary). The mean age was 23.6 years (SD 4.51; range 17-35 years). The study was approved by the behavioral research ethics board of the University of Saskatchewan. All participants provided written informed consent.

#### Power Analysis

A power analysis was carried out using G.Power 3.1.3 [84]. For a medium-sized effect (η²=0.15 or *f*=0.42), a medium-sized correlation between repeated measures, a power of 1–β=0.95, and an α value of .05, a minimum sample of 22 participants was needed to detect a significant difference between performance or subjective experience. Thus, failure to find a significant effect will support the null hypothesis.

#### SSG Design

**Figure 1:**
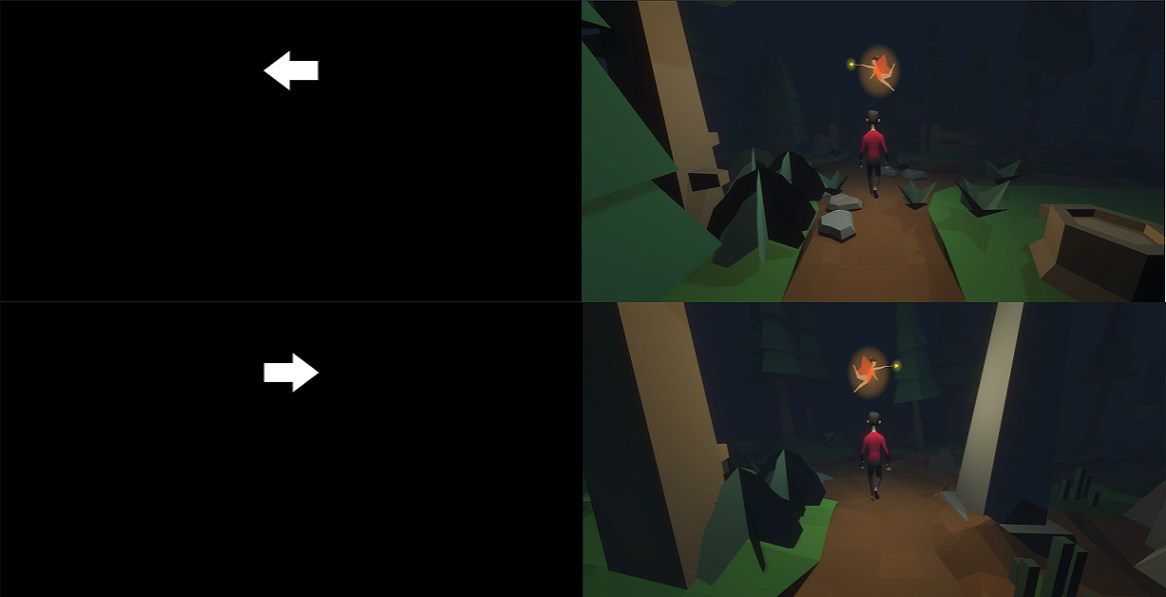
The stop-signal task (left) vs stop-signal game (right) trial appearance.

Both the SST and SSG were implemented using the Unity3D engine. The basic SST as well as the SSG consisted of 3 blocks, each containing 100 trials, 75% of which were go-trials and 25%, stop-trials. Between separate blocks, a pause of 15 seconds was granted. The go-stimulus was presented for a maximum of 1500 msec or until reaction. The stop signal was played over headphones following a variable delay (SSD), which was initially set to 250 msec. The SSD was continuously adjusted with the staircase procedure to obtain a probability of responding to 50%. After the reaction was successfully stopped (ie, button press was inhibited), the SSD was increased by 50 msec, whereas when the participants did not stop successfully, the SSD was decreased by 50 msec. The intertrial interval was set to a random value between 500 msec and 1500 msec. In the basic SST, participants had to respond to a left- or right-pointing arrow, which was presented in the upper third portion of the screen (Figure 1).

**Figure 2:**
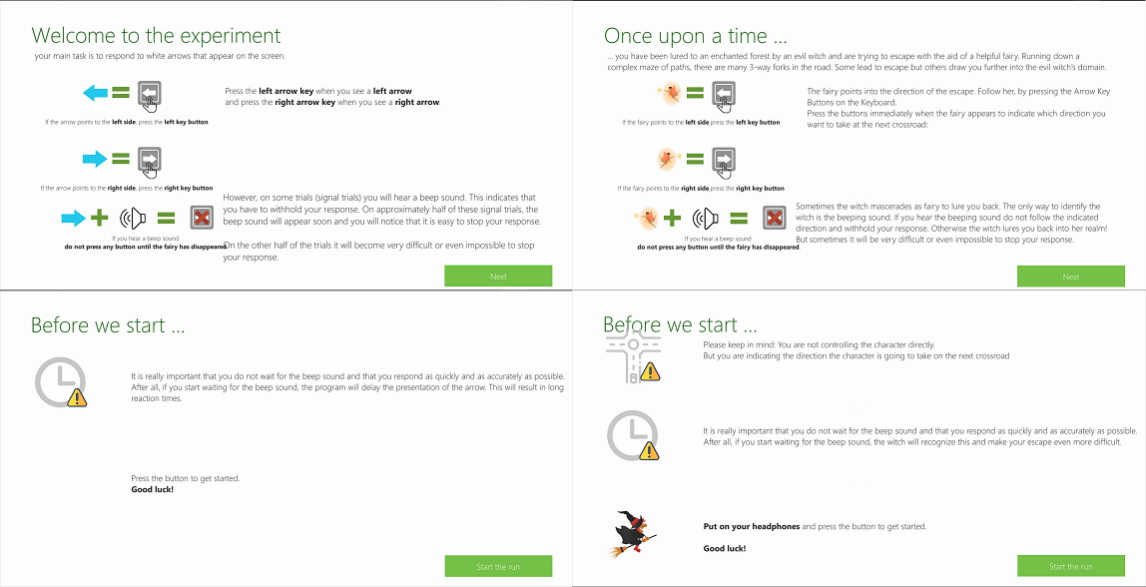
Instructions shown to participants for the stop-signal task (left) and stop-signal game (right).

Our goals in designing the SSG were to make the game as identical to the task as possible, while also providing enjoyment. As many gamified cognitive tasks end up being experienced as disappointing in terms of enjoyment [24,28], we built our game around a popular game genre and ensured professional quality graphics. The SSG was built on the 3D infinite runner genre, in which the player sees a third-person view of their avatar running down a path (similar to the popular mobile game Temple Run by Imangi Studios). The game premise was integrated with the task instructions, as shown in Figure 2: “Once upon a time you have been lured to an enchanted forest by an evil witch are trying to escape with the aid of a helpful fairy.” In contrast to the arrows used in the SST, the SSG presented arrows in the form of a magical fairy who was pointing to the left or right and would guide them out of the forest (Figure 1). However, players were told that the evil witch sometimes masqueraded as the fairy, and the only way to know was through a beeping sound (ie, the auditory stop signal); in this case, they were to withhold their response or be lured deeper into the forest. After a choice was made, the avatar turned in the direction selected by the player, regardless of whether or not it was correct (technically, the camera rotated the world and the avatar continued straight). If they failed to respond or correctly withheld their response, the avatar continued on straight. Each choice occurred at a crossroad so that all options were possible, regardless of player response. The terrain was procedurally generated and shaded so that the forest was very dark (matching the dark background of the SST). As shown in Figure 1, we used “low poly” game art, which refers to meshes in 3D computer graphics that contain a small number of polygons, to give a professional appearance in real time apps (ie, games), while optimizing performance.

The task was implemented using the Game Engine by Unity3D (version 2019.01) in a single version with a toggle button to switch between the SST and SSG (to keep the implementation of the underlying task identical). The differences between the SST and the SSG were the inclusion of a narrative theme and premise and the presence of the graphical elements, which included the background, the player avatar, and the stimulus (arrow or pointing fairy). The pointing fairy was designed to make the direction easily discriminable to avoid effects from overhead movement interfering with processing the intended movement direction, as replacing basic stimuli with more visually complex ones has been suggested to influence cognitive task performance [28]. In terms of gamification elements employed, we did not use points, scores, or a win or loss condition in the SSG but employed narrative elements including a backstory, a theme, and characters along with immersive elements of a 3D world and theme-appropriate graphics.

#### Stimuli and Apparatus

Participants were seated in front of a 27-inch color monitor with a viewing distance of approximately 80 cm. The study took place in an ordinarily lit room, 1 participant at a time. Participants were tasked to respond to the signals on screen by using 2 marked keys on the keyboard and withhold their response when an auditory stop signal (900 Hz) was presented over headphones. Participants were instructed to react as fast and accurately as possible. They were tasked to complete both SSTs: the basic SST and the SSG, as previously described. Both SST and SSG took approximately 15 min to complete.

#### Questionnaire Measures

A total of 2 established questionnaires were used to assess participant experience. The IMI measures motivation on 4 different subscales: interest-enjoyment, effort-importance, perceived competence, and tension-pressure. Each item was rated through an agreement with a statement on a 7-point scale (higher=greater agreement). The FSS assesses the subjective flow experience and factors influencing it using 9 subscales: challenge-skill balance, action-awareness merging, clear goals, unambiguous feedback, concentration on the task at hand, paradox of control, loss of self-consciousness, transformation of time, and autotelic experience. The FSS items were measured through an agreement with statements on a 5-point scale (higher=greater agreement).

#### Procedure

Participants were tasked to complete both the basic SST and the SSG, each followed immediately by the FSS and the IMI. The order of presentation of the SST and SSG was counterbalanced across participants and included as a factor in the analysis. After completion of both tasks and questionnaire sets, participants completed a demographic questionnaire.

#### Design

The study was based on a 2 (task: SST, SSG) x 2 (task-order: SST-SSG vs SSG-SST) mixed measures design with task as a within-subjects factor and task-order as a between-subjects factor.

#### Data Exclusion

In the data reduction phase, participants were excluded if they were uncooperative or produced faulty data. Initially, it was checked that all participants had normal or corrected-to-normal vision and hearing. For participant exclusion based on the SST and SSG performance, we followed the recommendations in the literature [85,86] that the SSRT can be reliably estimated for each participant in both sessions. Specifically, p(response|signal) had to be .4-.6, the horse-race model had to be satisfied, and the participant should not display strategic behavior (eg, waiting for the stop signal to appear). Furthermore, outliers based on the Tukey outlier criterion [87] within the data were identified and removed if necessary. After these procedures, 6 participants were excluded resulting in a sample of 24 with valid behavioral data. Furthermore, 1 additional participant had to be removed from the questionnaire analysis owing to a data collection error.

#### Dependent Measures

The main dependent variable for performance was the SSRT, that is, the estimate of time needed to respond to the stop signal and to cancel the movement, which measures the covert inhibition process. The estimation of the SSRT was based on the integration method with the replacement of omissions [85,88]. We also measured the SSD, the overall reaction time (RT) for both signal and no-signal trials, the probability of correct inhibition (p(response|signal)), and the omission and commission errors, as standard measures within the stop-signal paradigm [85]. The main dependent variables for experience were measured by using the IMI and FSS, as previously described.

#### Hypotheses

We hypothesized that there would be no difference in performance measures between the SSG and SST. Questionnaire data were analyzed to test our hypothesis that the SSG would elicit a more positive subjective experience as compared with the basic SST in terms of motivation and flow.

### Study 2: Between-Subject Design

#### Sample

A total of 39 healthy subjects (20 female and 19 male) aged between 18 and 36 years (mean age 24.26, SD 4.99 years) were recruited for the study. All participants had normal or corrected-to-normal hearing and vision. The study was approved by the behavioral research ethics board of the University of Saskatchewan. All participants provided written informed consent.

#### Eye Tracking

We used a 60 Hz Tobii 4C eye tracker to measure the user’s gaze focus. Areas of interest (AOIs) were mapped inside the app for subsequent analysis. The most important AOI was the instruction location (ie, stop-and-go signal location). For the SSG, additional AOIs were defined, including the path, the avatar, and the background.

#### Stimuli and Apparatus

These were identical as in study 1, apart from the eye-tracking device, which was mounted below the monitor.

#### Questionnaires

These were identical as in study 1.

#### Procedure

Upon entering the laboratory, participants were randomly divided into 2 groups: (1) basic SST and (2) gamified SSG. The eye tracker was calibrated for each participant; after calibration, participants started with the assigned task. After task completion, participants completed the questionnaires.

#### Design

The experiment was based on a two-group design. Each group was tasked to only complete either the basic SST or the gamified SSG.

#### Data Analysis

The experiment was based on a two-group (task: SSG vs SST) design. All other details are identical to study 1.

#### Data Exclusion

The procedure was identical to study 1. A total of 9 participants had to be excluded during the data reduction process resulting in a final sample of 30, evenly split between the 2 groups.

## Results

### Overview

A summary of the inference statistics in table form can be found in Multimedia Appendix 1. The tables (A1; stop-signal measures), A2 (IMI), and A3 (Flow) show means and SDs of measures in study 1, whereas tables A4 (stop-signal measures), A5 (IMI), and A6 (Flow) show the means and SDs for study 2.

### Study 1: Within-Subject Design

#### Performance Results

##### Control Analysis

It is recommended to validate the obtained stop-signal data by showing a significant difference between the average signal RT and the average no-signal RT, with higher RTs for no-signal trials. To this end, a 2 (task-order: SST-SSG vs SSG-SST) x 2 (task: SSG vs SST) x 2 (trial-type: signal vs no-signal) multivariate analysis of variance (MANOVA) was calculated. Only the main effect trial type (*F*_1,22_=300.38; *P*<.001; η²=0.92) was statistically significant, which shows that signal RT and no-signal RT were different in the expected direction. The main effect task (*F*_1,22_=2.55; *P*=.13) and the main effect order (*F*_1,22_=1.28; *P*=.27) were nonsignificant. Furthermore, all the two-way interactions and the three-way interactions did not yield a statistically significant result (all *F*<1).

##### SSRT

**Figure 3:**
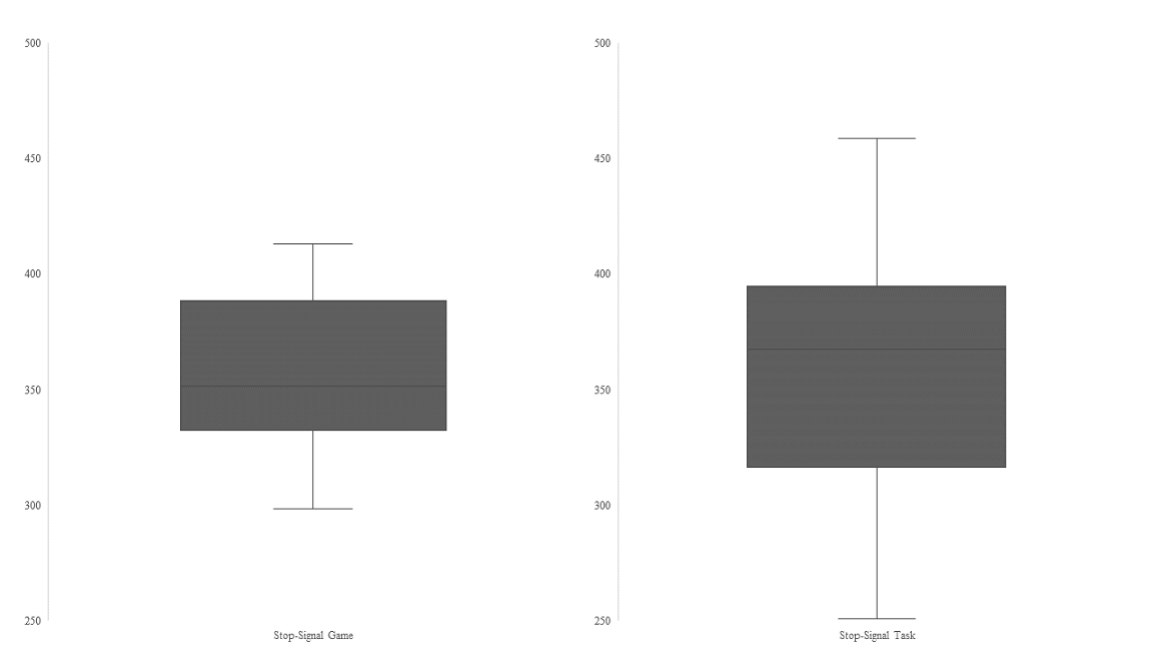
Stop-signal reaction time (in milliseconds) distribution depending on task type for study 1.

SSRT is an indirect estimate for the duration of the cognitive inhibition process, in which lower values represent higher inhibition speeds and efficiency. SSRTs were analyzed using a 2 (order: SST-SSG vs SSG-SST) x 2 (task: SSG vs SST) repeated measures MANOVA. The main effect task type (*F*_1,22_=0.03; *P*=.86) and the main effect order (*F*_1,22_=0.55; *P*=.47) as well as the interaction (*F*_1,22_=0.02; *P*=.88) were not significant. To illustrate the comparable SSRT values for both task types, Figure 3 shows the SSRT distribution depending on the task type. Thus, the speed of the inhibition process was not altered by any experimental manipulation, providing support for the equivalence of the 2 task types concerning their measurement properties (Table 1).

**Table 1:** Mean reaction time in milliseconds dependent on task type for study 1. The measurements are collapsed across the order of task performance.

Task	Signal reaction time	No-signal reaction time	Stop-signal delay	Stop-signal reaction time
Stop-signal task, mean (SD)	714.69 (164.83)	817.90 (181.60)	445.97 (185.87)	360.03 (53.86)
Stop-signal game, mean (SD)	750.67 (166.65)	851.55 (181.17)	476.02 (179.39)	358.33 (33.28)

##### SSD

A 2 (order: SST-SSG vs SSG-SST) x 2 (task: SSG vs SST) repeated measures MANOVA was computed. The main effect task type (*F*_1,22_=1.28; *P*=.27) and the main effect order (*F*_1,22_=1.51; *P*=.23) as well as the interaction (*F*_1,22_=0.02; *P*=.89) were not significant. Thus, there was no performance difference on the SSD depending on order or the type of SST (Table 1).

##### Signal RT

The incorrect signal RTs were analyzed using a 2 (order: SST-SSG vs SSG-SST) x 2 (task: SSG vs SST) repeated measures MANOVA. Neither the main effect task (*F*_1,22_=2.68; *P*=.12) nor the main effect order (*F*_1,22_=1.59; *P*=.22) or the interaction (*F*_1,22_=0.26; *P*=.31) were significant. This indicates that signal RT was not dependent on order or task type (Table 1).

##### No-Signal RT

Correct no-signal RTs were analyzed using a 2 (order: SST-SSG vs SSG-SST) x 2 (task: SSG vs SST) repeated measures MANOVA. The main effect task (*F*_1,22_=2.18; *P*=.15), the main effect order (*F*_1,22_=1.02; *P*=.33), and the interaction (*F*_1,22_=0.004; *P*=.95) were all not significant. This result illustrates that overall correct RTs were not dependent on order or task type (Table 1).

##### Correct Inhibition

The probability of correctly inhibiting a response (p(response|signal)) was analyzed using a 2 (order: SST-SSG vs SSG-SST) x 2 (task: SSG vs SST) repeated measures MANOVA. The main effect task type (*F*_1,22_=0.01; *P*=.93) and the main effect order (*F*_1,22_=1.10; *P*=.31) as well as the interaction (*F*_1,22_=0.01; *P*=.93) were not significant. Thus, there was no performance difference in correct inhibition depending on order or the type of SST employed (Table 2).

**Table 2:** Mean error rates and accuracy in their relative proportion to the total trial count dependent on task type for study 1. The measurements are collapsed across the order of task performance.

Task	p(response|signal)	Omission error	Choice error
Stop-signal task, mean (SD)	0.47 (0.03)	0.04 (0.074)	0.0043 (0.007)
Stop-signal game, mean (SD)	0.47 (0.03)	0.016 (0.023)	0.0024 (0.0046)

##### Error Analysis

A total of 2 types of errors can be made during go-trials: omission errors (ie, missing a response) and commission errors (ie, choosing the wrong directional reaction). Both were analyzed using 2 (order: SST-SSG vs SSG-SST) x 2 (task: SSG vs SST) repeated measures MANOVAs. For omission errors, the main effects task (*F*_1,22_=3.40; *P*=.08), the main effect order (*F*_1,22_=0.10; *P*=.76) and the two-way interaction (*F*_1,22_=0.61; *P*=.44) were not significant. Similarly, the main effect task (*F*_1,22_=2.85; *P*=.11) and the main effect order (*F*_1,22_=0.69; *P*=.42) and the interaction (*F*_1,22_=0.22; *P*=.64) were not significant with regard to the commission errors. Taken together, order and task type did not influence error rates (Table 2).

#### Experience Results

##### IMI

As a first step, reliability scores for all 4 IMI subscales were calculated, using Cronbach alpha. The 4 subscales interest-enjoyment, perceived competence, effort-importance, and tension-pressure showed reliability scores of α_ie_=.90, α_pc_=.91, α_ei_=.82, and α_tp_=.70, which were deemed satisfactory. As a second step, all scores were analyzed using separate 2 (task: SSG vs SST) x 2 (order: SST-SSG vs SSG-SST) MANOVAs. For the subscale interest-enjoyment, a significant main effect of task was observed (*F*_1,21_=16.35; *P*=.001; η²=0.44), whereas the main effect order (*F*_1,21_=0.03; *P*=.88) and the two-way interaction (*F*_1,21_=0.03; *P*=.86) were not significant. In detail, participants rated interest-enjoyment on average 0.8 points higher (SD 0.914) for the SSG compared with the SST. The type of task did not affect ratings on all other subscales. For perceived competence, the main effect task (*F*_1,21_=0.69; *P*=.41), the main effect order (*F*_1,21_=0.56; *P*=.46), and the two-way interaction (*F*_1,21_=0.81; *P*=.38) were not significant. For the subscale effort-importance, the main effect task (*F*_1,21_=0.02; *P*=.90) and the main effect order (*F*_1,21_=0.004; *P*=.95) as well as the interaction (*F*_1,21_=0.02; *P*=.90) were not statistically significant. Finally, the subscale tension-pressure was not modulated by task (*F*_1,21_=0.71; *P*=.71), order (*F*_1,21_=0.85; *P*=.37), or the interaction between the 2 variables (*F*_1,21_=1.10; *P*=.31). In summary, participants rated the game higher in interest-enjoyment compared with the basic version; the order in which tasks were completed did not affect the results. Owing to a lack of an overall scale score, the between-task difference values (Δ SSG-SST) were submitted to a multivariate analysis to determine whether or not overall IMI ratings differed among the tasks. The analysis revealed that when considering all subscales simultaneously, the SSG scored significantly higher compared with the SST (*F*_4,18_=6.35; *P*=.002; η²=0.59). Taken together, the analysis shows that the SSG scored significantly higher on the subscale interest-enjoyment (Cohen *d*=0.601) and was overall rated higher on the IMI (Cohen *d*=1.109). For scale means, refer to Table 3.

**Table 3:** Mean scale values for each Intrinsic Motivation Inventory subscale depending on task variant and the study (study 1).

Subscale	Stop-signal task, mean (SD)	Stop-signal game, mean (SD)
Interest-enjoyment	3.25 (1.45)	4.05 (1.20)
Perceived competence	3.83 (1.39)	3.99 (1.41)
Effort-importance	4.68 (1.39)	4.71 (1.32)
Tension-pressure	3.29 (1.17)	3.49 (1.11)

##### Flow

As a first step, reliability scores for all 9 flow subscales and the complete scale were calculated using the Cronbach alpha. The 9 subscales, challenge-skill balance (α_csb_=.85), action-awareness merging (α_awm_=.78), clear goals (α_cg_=.86), unambiguous feedback (α_uf_=.81), concentration on the task at hand (α_c_=.66), paradox of control (α_pc_=.90), loss of self-consciousness (α_lsc_=.82), transformation of time (α_tt_=.82), autotelic experience (α_ae_=.87), and the overall scale (α_overall_=.91), showed satisfactory reliability scores. As a second step, all scores were analyzed using separate 2 (task: SSG vs SST) x 2 (order: SST-SSG vs SSG-SST) MANOVAs. For the subscales challenge-skill balance, action-awareness merging, clear goals, paradox of control, loss of self-consciousness, and transformation of time, no significant effects emerged. In detail, with regard to the action-awareness merging subscale, the main effect task (*F*_1,21_=3.38; *P*=.08), the main effect order (*F*_1,21_=0.85; *P*=.36), and the interaction (*F*_1,21_=0.30; *P*=.59) were not significant. The main effects task (*F*_1,21_=0.48; *P*=.50), order (*F*_1,21_=0.04; *P*=.85), and their interaction (*F*_1,21_=0.001; *P*=.98) were not significant with regard to the action-awareness merging subscale. The analysis of the subscale clear goals neither revealed a significant main effect task (*F*_1,21_=2.24; *P*=.15) nor a main effect order (*F*_1,21_=0.06; *P*=.81) and no interaction (*F*_1,21_=0.16; *P*=.69). Neither the task type (*F*_1,21_=3.83; *P*=.06) nor the order (*F*_1,21_=0.32; *P*=.58,) or the interaction between task x order (*F*_1,21_=2.06; *P*=.17) were significant for the subscale paradox of control. Loss of self-consciousness was not modulated by the task (*F*_1,21_=1.72; *P*=.20) or by the order (*F*_1,21_=0.10; *P*=.76), and there was no interaction between the 2 variables (*F*_1,21_=2.73; *P*=.11). The ratings for transformation of time were neither influenced by the task (*F*_1,21_=0.14; *P*=.71) nor by the order (*F*_1,21_=0.002; *P*=.96) or their interaction (*F*_1,21_=0.02; *P*=.91). All effects with regard to the subscale unambiguous feedback were significant. In detail, the main effect task (*F*_1,21_=5.76; *P*=.04; η²=0.22) and the interaction of task x order (*F*_1,21_=5.76; *P*=.04; η²=0.22) displayed equally large effects whereas the effect for the main effect order (*F*_1,21_=3.77; *P*=.06; η²=0.15) was slightly smaller. Taken together, these results show that unambiguous feedback was rated higher in the game version than the basic task and this effect was enhanced when participants first worked on the basic version and then played the game. The interaction of task x order for the concentration on the task at hand score was significant (*F*_1,21_=6.81; *P*=.02; η²=0.25) whereas the main effect task (*F*_1,21_=1.06; *P*=.31) and order (*F*_1,21_=0.11; *P*=.74) were not, showing that concentration decreased in the second session regardless of which task version was done first or second. For an autotelic experience, the main effect task (*F*_1,21_=6.79; *P=*.02; η²=0.24) was significant whereas the main effect order (*F*_1,21_=0.40; *P*=.53) and the interaction (*F*_1,21_=0.0002; *P*=.99) were not, showing that participants were more internally driven playing the game version over the basic version. Most importantly, the overall Flow scale score was significantly higher for the SSG compared with the SST as indicated by the main effect task (*F*_1,21_=5.92; *P*=.02; η²=0.22) and there was no main effect order (*F*_1,21_=0.54; *P*=.47) or an interaction between task x order (*F*_1,21_=0.14; *P*=.71). To summarize, concentration on the task at hand decreased in the second session, which can be attributed to fatigue. In addition, the experience of unambiguous feedback increased when participants did the basic task first and then played the game. This result illustrates the participants’ feelings that the performance feedback was better and more responsive in the game version compared with the basic task. Furthermore, the results show that the overall experience of flow and the autotelic experience in particular were rated higher in the gamified version of the task. For mean values of the scale, refer to Table 4.

**Table 4:** Mean scale values for each Flow subscale depending on the task variant for study 1.

Subscale	Stop-signal task, mean (SD)	Stop-signal game, mean (SD)
Challenge-skill balance	3.10 (.90)	3.28 (.81)
Action-awareness merging	3.38 (.75)	3.26 (.84)
Clear goals	3.89 (.71)	4.03 (.62)
Unambiguous feedback	3.37 (.73)	3.52 (.83)
Concentration on the task	3.04 (.80)	3.26 (.75)
Paradox of control	3.12 (.96)	3.32 (.88)
Loss of self-consciousness	3.16 (1.03)	3.35 (1.00)
Transformation of time	3.05 (1.01)	3.00 (.86)
Autotelic experience	2.43 (.84)	2.78 (.79)
Overall	3.17 (.54)	3.31 (.49)

#### Bayesian Analysis

We employed the Bayesian analysis to put our results to an additional test and support any eventual interpretation of our data. Task version difference scores were calculated for each dependent variable (ie, all performance measures and scale values) to reflect the difference between SST and SSG. The difference scores for all performance variables (eg, SSRT, interest-enjoyment, challenge-skill balance) were submitted to Bayesian paired sample *t* tests using JASP. For performance measures, two-tailed tests were used, and for questionnaires, one-tailed tests were used. We used a Cauchy prior distribution with r=0.707. This prior was chosen because it reflects the range of most psychological effects [89] but given our hypothesis of a nonsignificant difference between the 2 task variations, it is a somewhat conservative prior. For the behavioral performance measures, an unspecific alternate hypothesis was specified (H1: SST≠SSG), whereas for the questionnaire data, a hypothesis-conform alternate hypothesis was chosen (H1: SSG>SST). The Bayesian analysis showed that there were no performance differences between SST and SSG. In detail, results showed weak-to-moderate support for the null hypothesis, and H0 was up to 4.63 times as likely as the alternative hypothesis depending on the behavioral performance measure in question [90-92]. With regard to the IMI, the analysis revealed decisive evidence for the subscale interest-enjoyment (BF_10_=168.11), whereas for all other IMI subscales, the null hypothesis was more likely (BF_01_ ranging from 2.4 to 4.19). Flow analysis showed mostly indecisive BFs, but there was moderate support for H0 with regard to the 2 subscales, action-awareness merging (BF_01_=7.21) and transformation of time (BF_01_=5.91). In contrast, the analysis revealed moderate support for H1 with regard to the autotelic experience (BF_10_=7.61) and the overall flow experience (BF_10_=4.91; Table 5).

**Table 5:** The Bayes factor table for study 1 shown by BF01 and BF10.

Dependent measures		Study 1			
		BF_01_^a^		BF_10_	
**Behavioral Performance Measures^b^**					
	No-signal reaction time		1.72		0.58
	Signal reaction time		1.48		0.68
	Stop-signal reaction time		4.60		0.22
	Stop-signal delay		2.52		0.40
	Omission errors		1.16		0.86
	Choice errors		1.37		0.73
	p(response|signal)		4.63		0.22
**Intrinsic Motivation Inventory**					
	Interest-enjoyment		0.006		168.11
	Perceived competence		2.40		0.42
	Effort-importance		4.19		0.24
	Tension-pressure		2.44		0.41
**Flow State Scale**					
	Challenge-skill balance		0.60		1.68
	Action-awareness merging		7.21		0.14
	Clear goals		0.82		1.22
	Unambiguous feedback		0.51		1.96
	Concentration		1.34		0.75
	Paradox of control		0.66		1.52
	Loss of self-consciousness		1.65		0.61
	Transformation of time		5.91		0.17
	Autotelic experience		0.13		7.61
	Overall		0.20		4.92

^a^BF: Bayes factor.

^b^For the behavioral performance measures H0: SSG=SST and H1: SSG≠SST. For questionnaire measures H1: SSG>SST.

### Study 2: Between-Subject Design

#### Performance Measures

##### Control Analysis

To establish that signal RT and no-signal RT significantly differ from each other, a 2 (task: SSG vs SST) x 2 (trial-type: signal vs no-signal) MANOVA was calculated. Only the main effect trial type (*F*_1,28_=156.14; *P*<.001; η²=0.85) was statistically significant, which showed that signal RT and no-signal RT trials were different in the expected direction. The main effect of task (*F*_1,28_=0.14; *P*=.71) as well as the two-way interaction (*F*_1,28_=1.37; *P*=.25) were not significant.

##### SSD

There was no significant difference between the game and the basic version with regard to SSD (*F*_1,28_=0.00006; *P*=.99).

##### SSRT

**Figure 4:**
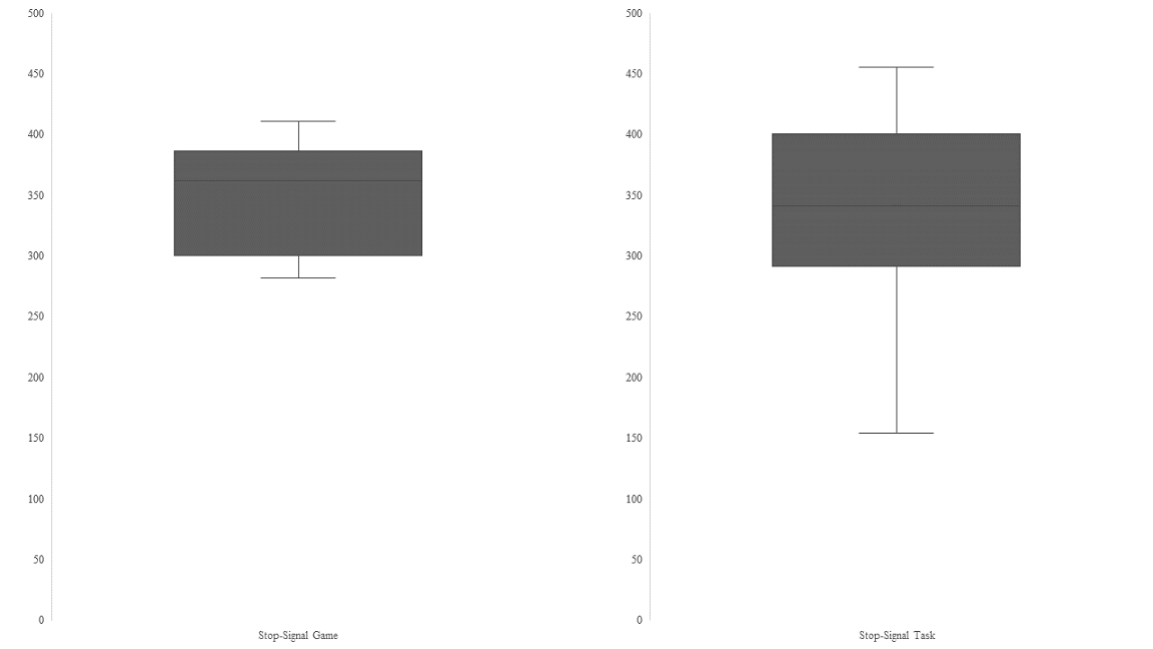
Stop-signal reaction time (in milliseconds) distribution depending on task type for study 2.

A one-way between-subjects analysis of variance (ANOVA) was conducted to compare the effect of task type (SSG vs SST) on SSRT. The main effect task (*F*_1,28_=0.03; *P*=.87) was not significant. Thus, the speed of the inhibition process did not depend on the task (Table 6; Figure 4).

**Table 6:** Mean reaction time in milliseconds dependent on the task type for study 2.

Task	Signal reaction time	No-signal reaction time	Stop-signal delay	Stop-signal reaction time
Stop-signal task, mean (SD)	648.12 (137.99)	759.90 (171.10)	406.03 (207.00)	344.33 (75.73)
Stop-signal game, mean (SD)	681.49 (183.49)	774.10 (204.26)	405.43 (208.52)	348.08 (46.11)

##### Signal RT

A one-way between-subjects ANOVA showed no difference between the game and basic version of the SST (*F*_1,28_=0.32; *P*=.58; Table 6).

##### No-Signal RT

Correct no-signal RTs did not differ between the task types (*F*_1,28_=0.04; *P*=.84; Table 6).

##### Correct Inhibition

The probability of correctly inhibiting a response (p(response|signal)) did not differ between the basic and game version (*F*_1,28_=0.79; *P*=.38; Table 7).

**Table 7:** Mean error rates and accuracy in their relative proportion to the total trial count dependent on the study and task type for study 2.

Task	p(response|signal)	Omission error	Choice error
Stop-signal task, mean (SD)	.49 (.04)	0.019 (0.034)	0.0021 (0.0038)
Stop-signal game, mean (SD)	.47 (.04)	0.020 (0.035)	0.0033 (0.0043)

##### Error Analysis

Neither the omission errors (*F*_1,28_=0.005; *P*=.94) nor the commission errors (*F*_1,28_=0.66; *P*=.42) differed between the 2 task versions. Taken together, order and task type did not influence error rates (Table 7).

##### Eye Tracking

**Figure 5:**
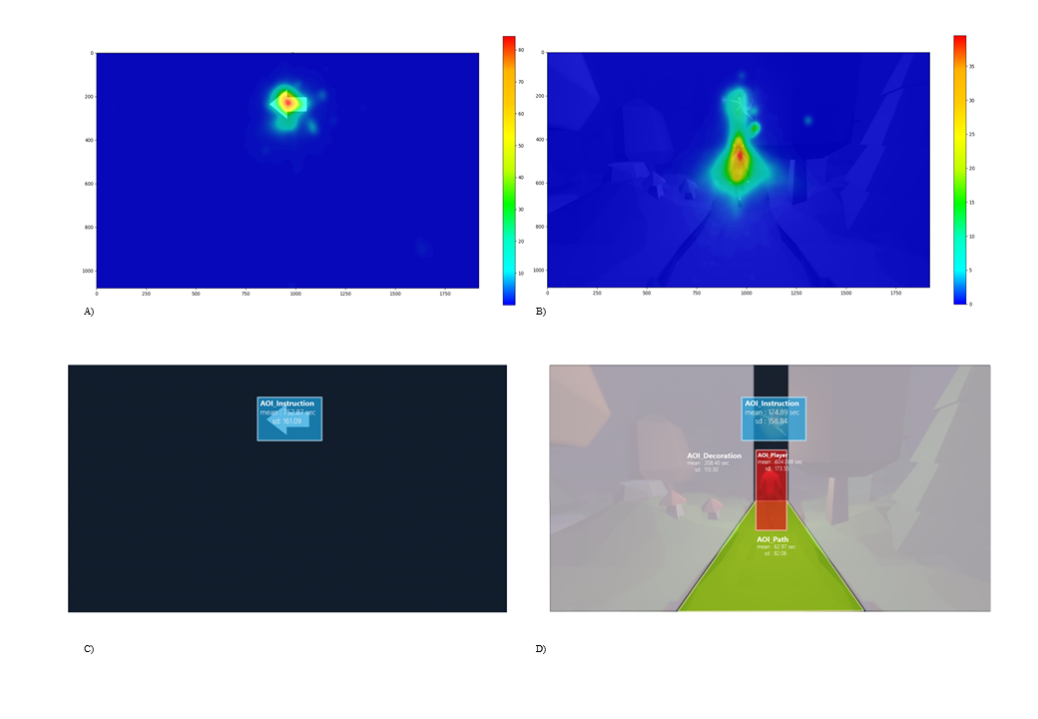
Visualization of the eye-tracking results. Parts (A) and (B) display the gaze focus as a heat map. Parts (C) and (D) display the mean time that the participants spent focused on parts of the display.

We recorded the estimated gaze fixation per user, which is the average fixation of the 2 eyes of the user. This screen coordinate was mapped on the previously introduced AOIs. Per user, we calculated the average focused time per AOI for both conditions over the complete experiment. In the gamified condition, users mostly focused on the avatar (mean 604.09 seconds, SD 173.55), less on the environment (mean 208.41 seconds, SD 113.31), and least on the instruction location (mean 174.89 seconds, SD 158.84), whereas they mostly looked at the instruction location in the basic version of the task (mean 752.88, SD 161.09). For an illustration of the results, see Figure 5.

#### Experience Measures

##### IMI

The reliability for all subscales was calculated. The 4 subscales interest-enjoyment, perceived competence, effort-importance, and tension-pressure showed reliability scores of α_ie_=.87, α_pc_=.89, α_ei_=.77, and α_tp_=.81, respectively. All subscale scores were submitted to a one-way between-subject ANOVA comparing the basic and the gamified task version. There were no significant differences for interest-enjoyment (*F*_1,28_=0.001; *P*=.98), perceived competence (*F*_1,28_=0.28; *P*=.60), effort-importance (*F*_1,28_=1.28; *P*=.27) and tension-pressure (*F*_1,28_=0.57; *P*=.46; Table 8).

**Table 8:** Mean scale values for each Intrinsic Motivation Inventory subscale depending on task variant and the study.

Subscale	Stop-signal task, mean (SD)	Stop-signal game, mean (SD)
Interest-enjoyment	3.09 (1.21)	3.11 (1.29)
Perceived competence	3.6 (1.29)	3.84 (1.19)
Effort-importance	4.3 (1.39)	4.78 (.89)
Tension-pressure	3.43 (1.44)	3.80 (1.22)

##### Flow

The reliabilities for all subscales and the overall reliability for the Flow scale was calculated. In detail, the 9 subscales, challenge-skill balance (α_csb_=.66), action-awareness merging (α_awm_=.66), clear goals (α_cg_=.71), unambiguous feedback (α_uf_=.74), concentration on the task at hand (α_c_=.82), paradox of control (α_pc_=.86), loss of self-consciousness (α_lsc_=.71), transformation of time (α_tt_=.79), autotelic experience (α_ae_=.88), and the overall scale (α_overall_=.86), showed satisfactory reliability scores. Overall, there were no statistical differences between the game and the basic version. In detail, the subscales for challenge-skill balance (*F*_1,28_=0.05; *P*=.83), action-awareness merging (*F*_1,28_=0.07; *P*=.80), clear goals (*F*_1,28_=3.20; *P*=.08), unambiguous feedback (*F*_1,28_=0.72; *P*=.40), the concentration on the task at hand (*F*_1,28_=3.27; *P*=.08), paradox of control (*F*_1,28_=1.36; *P*=.25), loss of self-consciousness (*F*_1,28_=1.56; *P*=.22), transformation of time (*F*_1,28_=0.65; *P*=.43), and autotelic experience (*F*_1,28_=0.01; *P*=.91) as well as all subscales combined (*F*_1,28_=0.83; *P*=.37) did not differ between the basic and game version (Table 9).

**Table 9:** Mean scale values for each Flow subscale depending on task variant and the study (study 2).

Subscale	Stop-signal task, mean (SD)	Stop-signal game, mean (SD)
Challenge-skill balance	3.08 (7.11)	3.13 (.52)
Action-awareness merging	3.20 (.71)	3.27 (.71)
Clear goals	3.93 (.50)	3.60 (.52)
Unambiguous feedback	3.47 (.77)	3.27 (.79)
Concentration on the task	3.17 (.78)	2.67 (.73)
Paradox of control	2.95 (.83)	3.28 (.73)
Loss of self-consciousness	3.53 (.92)	3.13 (.83)
Transformation of time	3.42 (.83)	3.18 (.75)
Autotelic experience	2.28 (.96)	2.32 (.59)
Overall	3.23 (.44)	3.09 (.34)

#### Bayesian Analysis

Similar to study 1, we tested the 2 stopping task types against each other using a Bayesian independent sample *t* test with the same parameters as in study 1. For the behavioral performance measures, an unspecific alternate hypothesis was specified (H1: SST≠SSG), whereas for the questionnaire data, a hypothesis-conform alternate hypothesis was chosen (H1: SSG>SST). We obtained moderate evidence for the null hypothesis with regard to the behavioral performance measures, confirming that there is no performance difference between the two. The analysis of IMI scores revealed no conclusive BFs and only a tendency toward the null hypothesis. Results of the FSS analysis showed moderate evidence for the null hypothesis in several subscales (ie, clear goals, unambiguous feedback, concentration, loss of self-consciousness, and transformation of time) as well as the overall scale score (Table 10).

**Table 10:** The Bayes factor (BF) table showing BF01 and BF10.

Dependent measures			Study 2		
			BF_01_^a^		BF_10_
**Behavioral Performance Measures** ^b^					
	No-signal reaction time	2.86		.35	
	Signal reaction time	2.57		.39	
	Stop-signal reaction time	2.87		.35	
	Stop-signal delay	2.90		.34	
	Omission errors	2.90		.35	
	Choice errors	2.26		.44	
	p(response|signal)	2.15		.47	
**Intrinsic Motivation Inventory**					
	Interest-enjoyment	2.85		.35	
	Perceived competence	1.94		.52	
	Effort-importance	1.08		.93	
	Tension-pressure	1.58		.63	
**Flow State Scale**					
	Challenge-skill balance	2.48		.40	
	Action-awareness merging	2.41		.42	
	Clear goals	6.83		.15	
	Unambiguous feedback	4.73		.21	
	Concentration	6.78		.15	
	Paradox of control	1.04		.97	
	Loss of self-consciousness	5.63		.18	
	Transformation of time	4.63		.22	
	Autotelic experience	2.68		.37	
	Overall	4.87		.21	

^a^BF: Bayes factor.

^b^For the behavioral performance measures H0: SSG=SST and H1: SSG≠SST. For questionnaire measures H1: SSG>SST.

## Discussion

### Principal Findings

Overall results show that our newly developed SSG can be used to measure response inhibition as well as SST while being more enjoyable. Specifically, in 2 studies employing a within-subject (study 1) and between-subject (study 2) design, we showed that there were no significant differences between the 2 tasks across all behavioral performance measures. Furthermore, we obtained strong evidence that the SSG was more enjoyable and led to higher experiences of flow but only when participants were able to compare the 2 tasks with each other.

In detail, the results of study 1 showed that performance did not differ between the SSG and the basic SST and that the order of tasks did not influence performance. Concerning the experience of flow and intrinsic motivation, the SSG was superior to the standard SST paradigm, with the largest effect being shown by the interest-enjoyment subscale in the IMI, in which 44% of the variance was explained by the game versus task manipulation. Importantly, effect sizes suggest the existence of a large difference between SST and SSG with regard to interest-enjoyment (η²=0.44; Cohen *d*=0.601) and overall intrinsic motivation (η²=0.59; Cohen *d*=1.109). Furthermore, the SSG scored higher on the flow subscales for an autotelic experience and unambiguous feedback, and the overall flow score was significantly higher for the SSG, with the game elements explaining 22% to 24% of the variance in experienced flow. These frequentist results were confirmed by the Bayesian analysis. First, there was evidence against performance differences between the 2 tasks. Second, we obtained decisive evidence for a higher interest-enjoyment rating in the SSG compared with the SST, whereas all other IMI subscales were not affected by the type of stopping task. Third, there was evidence for a higher level of autotelic and overall flow experience in the SSG compared with the SST. Overall, our findings suggest that the SSG can be used as a reliable measurement of the response inhibition process, while being experienced as more enjoyable for participants.

In a second study, we aimed to extend and replicate our findings. As there is evidence that stopping is influenced by perceptual distractors [83], we implemented an eye-tracking procedure to assess the gaze differences between the SST and the SSG. The eye-tracking implementation mirrors the exploratory analysis by Verbruggen et al [83]. Their exploratory analysis showed that the frequency of eye movements was increased in the condition where the stop signal was presented peripherally. If we had found a significant performance difference between the 2 task versions, we could have used the eye-tracking data to explain this result. On the contrary, our results show that despite a more visually complex environment, which modified gaze and eye movements, the SSG leads to a comparable performance with the SST. We opted for a between-subjects design, which has the additional benefit of eliminating any sequence effects on the eye-tracking data; especially, when people are aware that their behavior is tracked across different task versions, they might behave differently. With that being said, we expected the differences in subjective experience (ie, differences in questionnaire scores) to be smaller owing to the lack of a direct comparison in a between-subjects design.

The results of study 2 partially replicated the results of study 1. We did not find performance differences between SST and SSG in any performance measure. Contrary to study 1, an analysis of questionnaire scores showed that there was no difference between SST and SSG in either the IMI or Flow scale. A Bayes analysis confirmed these findings and revealed small-to-moderate evidence for the null hypothesis (H0: SST=SST) with regard to performance measures as well as the IMI and Flow scales. The lack of differences in questionnaire scores was somewhat expected. This result is likely due to the fact that participants in study 2 had no chance to compare the 2 task versions coupled with a regression toward the mean and a tendency of participants to avoid the more extreme scale ratings [93,94]. In addition, we think that the lack of a significant difference between tasks in study 2 is positive. It reflects that only when an implicit comparison between SST and SSG can be made are the 2 versions perceived differently, but overall, the influence of the gamification on motivation is not exceedingly large, as task performance was still comparable. Game elements that overly influence task performance can in turn make it difficult to gather an individual’s exact baseline performance. The average fixation time on the previously presented AOIs showed that the gaze focus differed between the 2 tasks. However, this crucially did not seem to affect performance. Interestingly, this hints at the possibility that foveal focus and attention is not required to effectively process a simple stop signal.

### Comparison With Previous Work

To the authors’ knowledge, there only has been one other study that tried to gamify the SST [30]. The aforementioned study compared 3 SST variants—standard, theme, and scoring—in participants who were recruited and tested on the web. They found no effect of task variant on attrition, and although the variant with the scoring system had higher ratings, the theme variant scored lower compared with the standard SST paradigm. Importantly however there are several differences between the study by Lumsden et al [30] and this paper. We employed the SST and SSG in a controlled lab environment and not on the web, which has the clear advantage of control over the environment, the experimental set-up, and participant compliance. Furthermore, Lumsden et al [30] focused on the rounds played by participants after 4 required initial sessions but found no effect of task-variant playtime. Although the amount played might be a good measure of motivation, the reward for playing was low—monetarily and intrinsically.

In detail, the gamification used in the study by Lumsden et al [30] consisted of a scoring system without any graphical changes to the task or a thematic variation of the SST; in this case, the player had to sort fruit into different buckets. The theme version of the SST did not implement a scoring system, which is similar to the SSG in this study. Although this was not tested and is pure speculation, the authors of this study think it is reasonable to assume that the haunted forest cover story provides a higher sense of urgency and might be more engaging than sorting fruit by color. Furthermore, Lumsden et al [30] did award participants with only 50 cents for every session after the fourth session, which may not have been enough to keep players motivated. With that being said, the authors mirrored our results by showing that there were no performance differences between the task versions. We decided against the more volatile measure of play sessions and aimed to directly capture performance and motivation. Nevertheless, we think that the study provided important initial evidence and that it might be interesting in the future to validate our SSG on the web.

### Limitations

This study has 3 important limitations. First, overall reaction times and inhibition speeds were elevated in both the SST and SSG compared with the ordinarily observed values [65,66,83,85,86,95]. Furthermore, there is evidence that SSRT is unaffected by the demands of the go task [95], but this is still up for debate [83,86]. However, RTs as reported in this study are not completely unusual, and as both tasks produced comparable performance measures, this elevation might be traced back to the samples. Second, we only found a reliable difference in flow and motivation between SSG and SST in study 1 (ie, within-subject design). As task-order did not affect the evaluation of SST or SSG, we speculate that the increased motivation and flow experience in the within-subject study was because both tasks could be compared side-by-side. This illustrates that those kinds of subjective questionnaire measures are somewhat context-dependent. Third, one could also take the neuroscientific approach to compare SST and SSG. In detail, if our claim is that the SSG is a methodologically valid and more enjoyable substitute of the SST that can measure response inhibition accurately, then similar neural correlates should be obtainable. Specifically, similar to the SST, we would expect the right prefrontal cortex to play a crucial role in stopping performance during the SSG and, in contrast to the SST, other areas more responsible for visual information processing should show increased activity during the SSG [65,66,96-100]. In addition, recent evidence suggests that performance differences in video games translate to differential brain activity [101]. Thus, future neuropsychological studies might have to take the individual baseline performance and brain activity into account.

### Outlook

There are several directions in which future research could be taken. As already mentioned in the introductory section of this paper, the ability to inhibit an already initiated action is linked to mental health conditions such as ADHD, OCD, schizophrenia, and posttraumatic stress disorder [59,69,102,103]. As video games are accessible, motivating, and can be custom built to capture behavior, such as the SSG in this paper, it has been proposed that digital games or game-like tasks can be useful for the assessment and treatment of mental health issues [104,105]. Although the use of cognitive psychological testing in a clinical setting is well established and those experimental approaches produce reliable between-group (ie, clinical vs nonclinical sample) differences, they are not necessarily reliable on an individual level over time [106]. Nevertheless, we propose that the SSG should be experimented with its use in a more applied setting. This can be especially important in cases where obtaining a valid response inhibition measurement is difficult. For example, in some clinical subsamples, the ability to focus on the task at hand is limited, and the SSG is more easily accessible and motivating for participants although validly measuring the stopping ability. To this end, a first step could be to validate the present results on a larger scale in a remote web-based assessment. A web-based assessment of behavioral as well as psychophysiological measures via game-like tasks has been done before and shown to be promising for the future [107,108].

### Conclusions

Taken together, our results suggest that the newly developed SSG is an effective tool to measure the response inhibition process. The SSG compared with the regular SST has 2 clear advantages. First, the SSG leads to higher enjoyment and flow, and second, it assesses an individual’s stopping capabilities in a more realistic, ecologically valid setting.

## Appendix

Multimedia Appendix 1 Summary of the performance analysis results.

## Abbreviations

ADHD: attention-deficit/hyperactivity disorder
ANOVA: analysis of variance
AOI: area of interest
BF: Bayes factor
FSS: Flow State Scale
IMI: Intrinsic Motivation Inventory
MANOVA: multivariate analysis of variance
OCD: obsessive-compulsive disorder
RT: reaction time
SDT: Self-Determination Theory
SSD: stop-signal delay
SSG: stop-signal game
SSRT: stop-signal reaction time
SST: stop-signal task
