# Supplementary material for: Effective Gamification of the Stop-Signal Task: Two Controlled Laboratory Experiments
Source: JMIR Serious Games. 2020 Sep 8;8(3):e17810. doi: 10.2196/17810 (PMC7509611; doi:10.2196/17810)
Supplement: Multimedia Appendix 1 [file games_v8i3e17810_app1.docx]

## Appendix

Table A1. Summary of the performance analysis results for study 1. η² is only provided for statistically significant results.

|  | effect | F(df) | p | η²_p_ |
| --- | --- | --- | --- | --- |
| control analysis | | | | |
|  | main effect trial-type | *F*(1, 22) = 300.38 | <. 0001 | .92 |
|  | main effect task | *F*(1, 22) = 2.55 | .13 |  |
|  | main effect order | *F*(1, 22) = 1.28 | .27 |  |
|  | task x trial-type | *F*(1, 22) = .10 | .76 |  |
|  | task x order | *F*(1, 22) = .88 | .77 |  |
|  | order x trial-type | *F*(1, 22) = .52 | .48 |  |
|  | task x trial-type x order | *F*(1, 22) = .98 | .33 |  |
| SSRT | | | | |
|  | main effect task | *F*(1, 22) = .03 | .86 |  |
|  | main effect order | *F*(1, 22) = .55 | .47 |  |
|  | order x task | *F*(1, 22) = .02 | .88 |  |
| SSD | | | | |
|  | main effect task | *F*(1, 22) = 1.28 | .27 |  |
|  | main effect order | *F*(1, 22) = 1.51 | .23 |  |
|  | order x task | *F*(1, 22) = .02 | .89 |  |
| p(response\|signal) | | | | |
|  | main effect task | *F*(1, 22) = .01 | .93 |  |
|  | main effect order | *F*(1, 22) = 1.10 | .31 |  |
|  | order x task | *F*(1, 22) = .01 | .93 |  |
| omission error | | | | |
|  | main effect task | *F*(1, 22) = 3.40 | .08 |  |
|  | main effect order | *F*(1, 22) = .10 | .76 |  |
|  | order x task | *F*(1, 22) = .61 | .44 |  |
| commission error | | | | |
|  | main effect task | *F*(1, 22) = 2.85 | .11 |  |
|  | main effect order | *F*(1, 22) = .69 | .42 |  |
|  | order x task | *F*(1, 22) = .22 | .64 |  |
| signal RT | | | | |
|  | main effect task | *F*(1, 22) = 2.68 | .12 |  |
|  | main effect order | *F*(1, 22) = 1.59 | .22 |  |
|  | order x task | *F*(1, 22) = .26 | .31 |  |
| no-signal RT | | | | |
|  | main effect task | *F*(1, 22) = 2.18 | .15 |  |
|  | main effect order | *F*(1, 22) = 1.02 | .33 |  |
|  | order x task | *F*(1, 22) = .004 | .95 |  |

Table A2. Intrinsic-motivation-inventory (IMI) analysis results for study 1. η² is only provided for statistically significant results.

|  | effect | F(df) | p | η²_p_ |
| --- | --- | --- | --- | --- |
| interest-enjoyment | | | | |
|  | main effect task | *F*(1, 21) = 16.35 | < .01 | .44 |
|  | main effect order | *F*(1, 21) = .03 | .88 |  |
|  | order x task | *F*(1, 21) = .03 | .86 |  |
| perceived competence | | | | |
|  | main effect task | *F*(1, 21) = .69 | .41 |  |
|  | main effect order | *F*(1, 21) = .56 | .46 |  |
|  | order x task | *F*(1, 21) = .81 | .38 |  |
| effort-importance | | | | |
|  | main effect task | *F*(1, 21) = .02 | .90 |  |
|  | main effect order | *F*(1, 21) = .004 | .95 |  |
|  | order x task | *F*(1, 21) = .02 | .90 |  |
| tension-pressure | | | | |
|  | main effect task | *F*(1, 21) = .71 | .71 |  |
|  | main effect order | *F*(1, 21) = .85 | .37 |  |
|  | order x task | *F*(1, 21) = 1.10 | .31 |  |
| overall | | | | |
|  | main effect task | *F*(4, 18) = 6.35 | < .01 | .59 |

Table A3. Flow-state-scale (FSS) analysis results for study 1. η² is only provided for statistically significant results.

|  | effect | F(df) | p | η²_p_ |
| --- | --- | --- | --- | --- |
| challenge-skill balance | | | | |
|  | main effect task | *F*(1, 21) = 3.38 | .08 |  |
|  | main effect order | *F*(1, 21) = .85 | .36 |  |
|  | order x task | *F*(1, 21) = .30 | .59 |  |
| action-awareness merging | | | | |
|  | main effect task | *F*(1, 21) = .48 | .50 |  |
|  | main effect order | *F*(1, 21) = .04 | .85 |  |
|  | order x task | *F*(1, 21) = .001 | .98 |  |
| clear goals | | | | |
|  | main effect task | *F*(1, 21) = 2.24 | .15 |  |
|  | main effect order | *F*(1, 21) = .06 | .81 |  |
|  | order x task | *F*(1, 21) = .16 | .69 |  |
| unambiguous feedback | | | | |
|  | main effect task | *F*(1, 21) = 5.76 | < .05 | .22 |
|  | main effect order | *F*(1, 21) = 3.77 | < .05 | .15 |
|  | order x task | *F*(1, 21) = 5.76 | < .05 | .22 |
| concentration on the task at hand | | | | |
|  | main effect task | *F*(1, 21) = 1.06 | .31 |  |
|  | main effect order | *F*(1, 21) = .11 | .74 |  |
|  | order x task | *F*(1, 21) = 6.81 | < .05 | .25 |
| paradox of control | | | | |
|  | main effect task | *F*(1, 21) = 3.83 | .06 |  |
|  | main effect order | *F*(1, 21) = .32 | .58 |  |
|  | order x task | *F*(1, 21) = 2.06 | .17 |  |
| loss of self-consciousness | | | | |
|  | main effect task | *F*(1, 21) = 1.72 | .20 |  |
|  | main effect order | *F*(1, 21) = .10 | .76 |  |
|  | order x task | *F*(1, 21) = 2.73 | .11 |  |
| transformation of time | | | | |
|  | main effect task | *F*(1, 21) = .14 | .71 |  |
|  | main effect order | *F*(1, 21) = .002 | .96 |  |
|  | order x task | *F*(1, 21) = .02 | .91 |  |
| autotelic experience | | | | |
|  | main effect task | *F*(1, 21) = 6.79 | < .05 | .24 |
|  | main effect order | *F*(1, 21) = .40 | .53 |  |
|  | order x task | *F*(1, 21) = .0002 | .99 |  |
| overall |  |  |  |  |
|  | main effect task | *F*(1, 21) = 5.92 | < .05 | .22 |
|  | main effect order | *F*(1, 21) = .54 | .47 |  |
|  | order x task | *F*(1, 21) = .14 | .71 |  |

Table A4. Performance analysis results for study 2. η² is only provided for statistically significant results.

|  | effect | F(df) | p | η²_p_ |
| --- | --- | --- | --- | --- |
| control analysis | | | | |
|  | main effect trial-type | *F*(1, 28) = 156.14 | <. 001 | .85 |
|  | main effect task | *F*(1, 28) = .14 | .71 |  |
|  | main effect order | *F*(1, 28) = 1.37 | .25 |  |
| SSRT | | | | |
|  | main effect task | *F*(1, 28) = .03 | .87 |  |
| SSD | | | | |
|  | main effect task | *F*(1, 28) = .00006 | .99 |  |
| p(response\|signal) | | | | |
|  | main effect task | *F*(1, 28) = .79 | .38 |  |
| omission error | | | | |
|  | main effect task | *F*(1, 28) = .005 | .94 |  |
| commission error | | | | |
|  | main effect task | *F*(1, 28) = .66 | .42 |  |
| signal RT | | | | |
|  | main effect task | *F*(1, 28) = .32 | .58 |  |
| no-signal RT | | | | |
|  | main effect task | *F*(1, 28) = .04 | .84 |  |

Table A5. Intrinsic-motivation-inventory (IMI) analysis results for study 2. η² is only provided for statistically significant results.

|  | effect | F(df) | p | η²_p_ |
| --- | --- | --- | --- | --- |
| interest-enjoyment | | | | |
|  | main effect task | *F*(1, 28) = .001 | .98 |  |
| perceived competence | | | | |
|  | main effect task | *F*(1, 28) = .28 | .60 |  |
| effort-importance | | | | |
|  | main effect task | *F*(1, 28) = 1.28 | .27 |  |
| tension-pressure | | | | |
|  | main effect task | *F*(1, 28) = .57 | .46 |  |

Table A6. Flow-state-scale (FSS) analysis results for study 2. η² is only provided for statistically significant results.

|  | effect | F(df) | p | η²_p_ |
| --- | --- | --- | --- | --- |
| challenge-skill balance | | | | |
|  | main effect task | *F*(1, 28) = .05 | .83 |  |
| action-awareness merging | | | | |
|  | main effect task | *F*(1, 28) = .07 | .80 |  |
| clear goals | | | | |
|  | main effect task | *F*(1, 28) = 3.20 | .08 |  |
| unambiguous feedback | | | | |
|  | main effect task | *F*(1, 28) = .72 | .40 |  |
| concentration on the task at hand | | | | |
|  | main effect task | *F*(1, 28) = 3.27 | .08 |  |
| paradox of control | | | | |
|  | main effect task | *F*(1, 28) = 1.36 | .25 |  |
| loss of self-consciousness | | | | |
|  | main effect task | *F*(1, 28) = 1.56 | .22 |  |
| transformation of time | | | | |
|  | main effect task | *F*(1, 28) = .65 | .43 |  |
| autotelic experience | | | | |
|  | main effect task | *F*(1, 28) = .01 | .91 |  |
| overall |  |  |  |  |
|  | main effect task | *F*(1, 28) = .83 | .37 |  |
